# Building test data from real outbreaks for evaluating detection algorithms

**DOI:** 10.1371/journal.pone.0183992

**Published:** 2017-09-01

**Authors:** Gaetan Texier, Michael L. Jackson, Leonel Siwe, Jean-Baptiste Meynard, Xavier Deparis, Herve Chaudet

**Affiliations:** 1 Pasteur Center in Cameroun, Yaoundé, Cameroun; 2 UMR 912 / SESSTIM - INSERM/IRD/Aix-Marseille University / Faculty of Medicine - 27, Bd Jean Moulin, Marseille, France; 3 Group Health Research Institute, Seattle, United States of America; 4 Sub-Regional Institute of Statistics and Applied Economics (ISSEA), Yaoundé, Cameroun; 5 French Armed Forces Center for Epidemiology and Public Health (CESPA), Camp de Sainte Marthe, Marseille, France; Health Protection Agency, UNITED KINGDOM

## Abstract

Benchmarking surveillance systems requires realistic simulations of disease outbreaks. However, obtaining these data in sufficient quantity, with a realistic shape and covering a sufficient range of agents, size and duration, is known to be very difficult. The dataset of outbreak signals generated should reflect the likely distribution of authentic situations faced by the surveillance system, including very unlikely outbreak signals. We propose and evaluate a new approach based on the use of historical outbreak data to simulate tailored outbreak signals. The method relies on a homothetic transformation of the historical distribution followed by resampling processes (Binomial, Inverse Transform Sampling Method—ITSM, Metropolis-Hasting Random Walk, Metropolis-Hasting Independent, Gibbs Sampler, Hybrid Gibbs Sampler). We carried out an analysis to identify the most important input parameters for simulation quality and to evaluate performance for each of the resampling algorithms. Our analysis confirms the influence of the type of algorithm used and simulation parameters (i.e. days, number of cases, outbreak shape, overall scale factor) on the results. We show that, regardless of the outbreaks, algorithms and metrics chosen for the evaluation, simulation quality decreased with the increase in the number of days simulated and increased with the number of cases simulated. Simulating outbreaks with fewer cases than days of duration (i.e. overall scale factor less than 1) resulted in an important loss of information during the simulation. We found that Gibbs sampling with a shrinkage procedure provides a good balance between accuracy and data dependency. If dependency is of little importance, binomial and ITSM methods are accurate. Given the constraint of keeping the simulation within a range of plausible epidemiological curves faced by the surveillance system, our study confirms that our approach can be used to generate a large spectrum of outbreak signals.

## 1 Introduction

Many outbreak detection algorithms (ODA) for disease surveillance have been proposed in the literature (see for example Chen’s review [1]), and designers of epidemiological and laboratory surveillance systems need to opt for the most suitable.

The choice of an algorithm should be based on reproducible evaluations carried out under conditions as close to real life as possible [2]. Several authors including Buckeridge [3] and Jackson [4] have shown that algorithm evaluation depends on the characteristics of the outbreak curve (shape, duration and size) and the baseline (mean and variance). The evaluation process thus depends heavily on the quality of test data and the control of characteristics known to influence these results [5].

To evaluate an ODA, specific datasets composed of outbreak curves over a baseline are usually needed. These two components can be obtained from real data, synthetic data or from a combination of the two (semi-synthetic dataset), as described in Mandl [6] and Buckeridge [5].

The baseline is often obtained from the real, daily, routine data of disease surveillance systems, which are specific to the population under surveillance [3, 7]. Useful simulation tools also exist for simulating a baseline (such as Project Mimic, http://www.projectmimic.com/).

However, obtaining a sufficient quantity of outbreak curves with a realistic shape and covering a sufficient range of agents, size and duration, is known to be very difficult [3–5, 8]. With regard to disease outbreak curves, several authors have pointed out [3, 5, 7, 9] that the use of real data for outbreak signals is hindered by the shortage of real datasets that include enough clearly-defined outbreak signals. Natural outbreak signals cannot enable the control of features required for evaluating ODA (such as outbreak size, duration and shape). Buckeridge et al. [5] show that among 22 studies using natural outbreak signals, only five had enough outbreaks to evaluate and report quantitative measures such as sensitivity and timeliness. They conclude that evaluations based on natural signals should be reserved for qualitative evaluation. Similarly, Wagner [9] believes that a minimum of roughly 10 outbreaks is required to build a quantitative evaluation.

In contrast, synthetic outbreak curves have the potential to express the diversity of biological threats that a surveillance system could encounter and must detect. Synthetic datasets allow precise specification of the outbreak curve and make it possible to build large test sets. They are more convenient for sensitivity analyses and for exploring the effects of curve changes. Most evaluation studies in the last decade have used semi-synthetic datasets that combine real baseline data with synthetic outbreaks [5].

Several methods can be used to build synthetic outbreak curves. Mechanistic disease models, such as the Susceptible-Infected-Recovered (SIR) model, are commonly used [10, 11]. Agent-based models (ABM) such as EpiSims [12] build a network-based model of disease spread instead of a compartment-based one [9].

However, the purpose of most studies that simulate curves based on these approaches is not to generate outbreak curves to evaluate ODA, but is usually to predict an overall outbreak trend (until real-time quality forecasting is achieved and to identify realistic averaged parameters for describing outbreaks. For example, Nsoesie et al. [13] used an agent-based model, which requires the building offive preliminary models. They worked mainly on influenza outbreak forecasting and conceded the need for each surveillance team to build a social network model adapted to the population under surveillance.

One limitation of this type of approach is that a specific model must be constructed for each type of disease to be simulated [6, 13]. These models can be computationally expensive and data intensive, as well as difficult to set up for a non-specialist user. Building a realistic model can be a particularly challenging task due to the lack of knowledge and real data available for setting up model parameters and for the validation process [14]. Held [15] considers such mechanistic modeling as too ambitious to be used routinely in a disease surveillance system, pointing out that the non-availability of certain information (number of susceptible people, model parameters, etc.) renders the process unfeasible. In addition, these models are usually based on multiple assumptions that may be difficult to verify [9]. Due to the high level of statistical and mathematical modeling needed and according to our experience of disease surveillance system deployment, we are not convinced that the majority of users in charge of disease surveillance systems are able to generate curves with this approach and wish to do it.

A simpler approach is to build the synthetic outbreak curve by using a simple mathematical distribution that describes its expected shape. Uniform, linear, exponential, sigmoid, lognormal or Dirac distributions are often used [6, 7, 16–19] (S1 Fig). However, estimating the behavior of an ODA for real outbreaks from simple synthetic signals is not straightforward. The principal limitation of evaluations using semi-synthetic datasets is that they only find the smallest peak that could be detected, leaving the critical question of detectability unanswered: “What is the smallest outbreak that can be detected?”. We have known since Wagner[9], Buckeridge [3] and Jackson [20] that the answer is influenced by the outbreak shape for the selected size and duration, leading us to believe that ODA evaluations based solely on the injection of geometric shape can only be considered as partial evaluations.

Bollen [21] considers that a curve reflects latent determinants, which are hidden. Baker [22] and Tucker [23] also consider that observations of repeated measures over time are the result of latent factors and characteristics that can only be indirectly observed using real curves. Similarly, we may consider that a curve shape depicts a type of disease outbreak determined by a transmissible agent, a population, an environment and a collecting structure, which are the latent determinants. To improve the realism of produced curves, some authors propose that the modeling process be extended, by taking into consideration the effects of disease surveillance systems. In the case of the statistical distribution simulation approach, Lotze and coll. [7] proposed that system effect (day of the week, seasonal and holiday effect) be added to initial outbreak lognormal signatures.

Despite this improvement, the traditional methods (mechanistic or statistical distribution-based methods) for obtaining synthetic epidemic curves remain, for the most part, sophisticated or poorly realistic.

**Fig 1:**
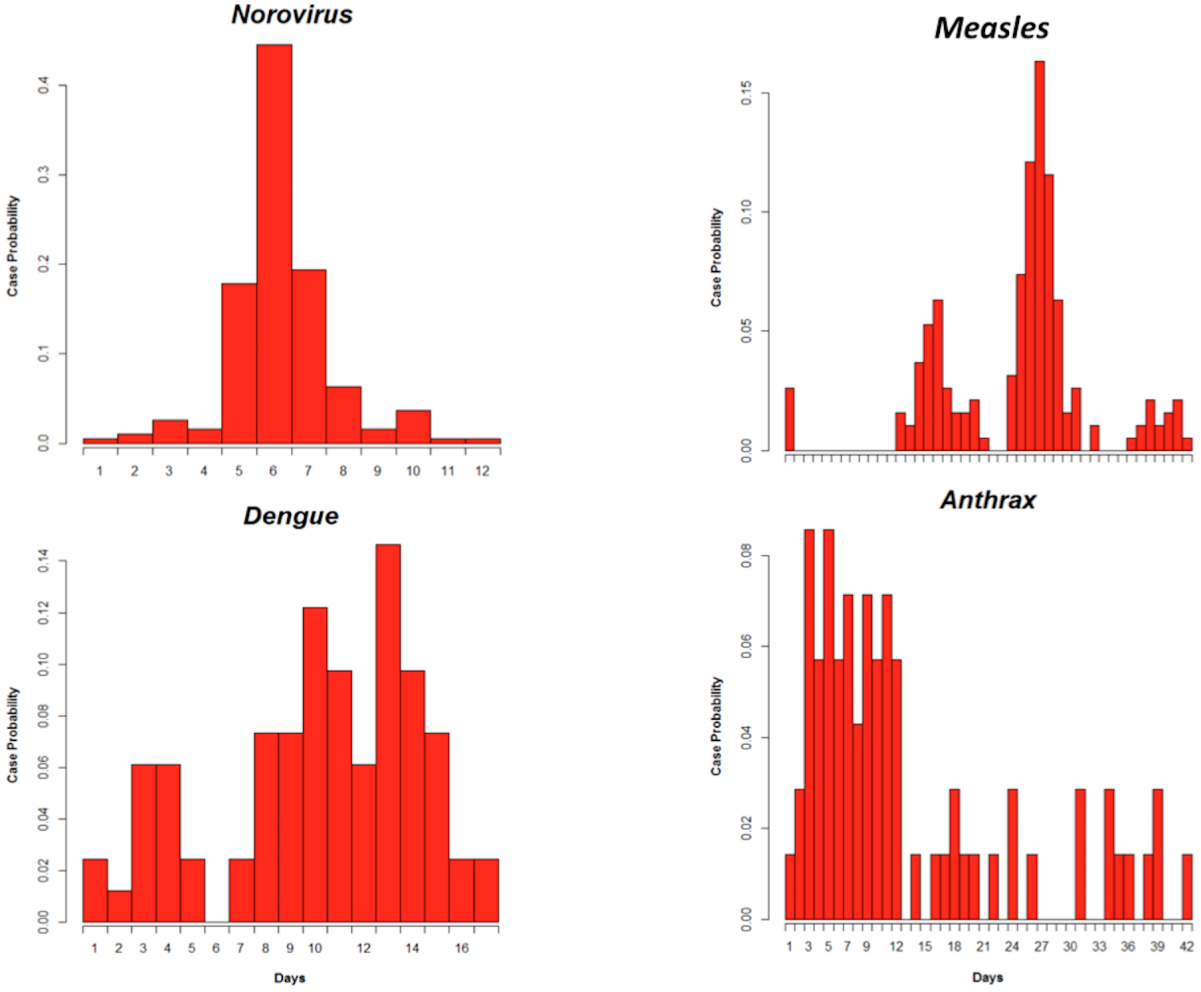
Probability mass function (PMF) for four published epidemic curves (norovirus, measles, anthrax and dengue outbreaks) represented by the case probability according to the time step of the outbreak [30–33].

Finally, the main way to generate an outbreak curve is by using an explicit propagation model (ABM, SEIR, distribution modeling, etc.). Modeling this process and quantifying the relative weight of each known factor are valuable for understanding the mechanisms influencing the outbreak dynamics. But all this modeling process is then required even if the final goal of this simulation is not to understand the epidemiological mechanisms producing the curve or to implement an explicative/forecasting model of the outbreak. Because our main goal here is to produce a realistic yet customized curve for a pathogen in a large range of surveillance situations, the relevance of model-based simulation for this specific purpose must be questioned. Indeed, there is a risk of generating only generalist or averaged curves that can be non-representative of the wide variety of surveillance curves, and the simulation process may suffer from a lack of realism. For example, the “classic” norovirus epicurve used in our study (Fig 1) [24] should be simulated by a Gaussian model, but is not able to represent the “atypical” epicurve observed in a specific population during a cruise [25]. Outbreak modeling is known to be highly complex [26], covering highly variable outbreak expressions and requiring numerous hypotheses and parameters. Taking an extreme position, some authors consider that carrying out realistic outbreak modeling is risky [27, 28]. As explained before, a latent model probably exists where the population under surveillance, the pathogens, the surveillance system and many other factors closely interact. An epicurve can be seen as the directly observable result of all these co-acting factors in association with unknown co-factors. We also need to take into consideration the risk of model misspecification, which may produce unrealistic or inaccurate curves. This topic is developed in the discussion proposed by Nishiura [29] and Horner [30], associated with a possible misspecification of the lognormal model proposed by Sartwell in 1966 [31] and largely used by the community. If we adopt this approach to consider the cruise outbreak, Gaussian simulation of all norovirus curves should be considered as a misspecification of the “atypical” curve (more adequately represented by an exponential decay or a Gamma model with different parameters).

Another possibility is to use simulation solutions that do not require modeling beforehand, such as Monte Carlo resampling methods. Indeed, these methods are tailored for simulating complex curves, especially when mathematical formalizations are not accessible, which is frequently the case for epidemic curves. S2 Fig describes what Monte Carlo resampling methods could add to the existing solutions used in the literature.

This study aims at verifying if a new and simple approach based on historical outbreak curves can be used to produce a range of outbreak signals (in terms of diversity of curve shapes) including very unlikely signals. The generated dataset of signals should reflect the likely distribution and the diversity of authentic signals faced by the surveillance system, while conserving as much as possible the shape realism that must conform to the apparent outbreak characteristics conveyed by the curve shape. To achieve this goal, we will evaluate if this new approach using homothetic transformation and resampling methods applied to historical disease outbreaks can be used to produce customized curves by controlling outbreak duration and size, while preserving curve shape (identified as critical for an ODA evaluation).

## 2 Material and methods

If we want to test algorithm ability and limitations under a large range of real surveillance situations, an adequate simulation tool must also be able to create the entire spectrum of outbreak curves, even those considered to be “atypical” but already-observed curves (qualified as “outliers”) must be considered, provided their atypical nature is recognized. The risk of a statistical modeling approach is that it might restrict the scope of the simulation used for the algorithm evaluation to an average behavior of the epidemics, which could be non-representative of the large range of shape varieties observed for the same pathogen. Using outlier curves precisely allows algorithm limitations to be evaluated against unusual outbreaks. This is why we chose real datasets from published disease outbreaks, representing different modes of transmission (Fig 1) and a range of shape varieties.

Probability mass functions (PMF) and cumulative distribution functions (CDF) will be extracted from the outbreak curves by computing the probability of the number of cases (or the cumulative probability) for each day of the epidemic. We propose that homothetic transformation (known to be a uniform scaling technique that preserves form) be used to control outbreak duration, together with a resampling method based on the Monte Carlo approach to manage its size. S2 Fig gives an example of the simulation and evaluation process (detailed above) in the case of a measles outbreak shape, for a 14-day duration and 30-case outbreak simulation.

We used R 3.0.1 (R Core team 2013) to implement all the methods described below [32].

### 2.1 Scaling outbreak duration

We apply a homothetic transformation to a PMF covering n time units to compute a new probability distribution lasting m time units. This transformation is a uniform dilation of the number of days using a scaling factor r, such as:

$$P_{j}=\overset{n}{\underset{i=1}{\sum }}f_{ij}.\pi_{i}\;\;\;\;\;\;with\;\;\;\;\overset{n}{\underset{i=1}{\sum }}\pi_{i}=1\;\;;\;\overset{m}{\underset{j=1}{\sum }}P_{j}=1\;;\;r=\;n/m$$

Where:

- *π*_*i*_ is the initial probability at time *i* in the PMF;
- *P*_*j*_ is the final probability at time j in the new probability distribution;
- *f*_*ij*_ is the fraction of π_i_ at time i participating in P_j_ and depending on r = n/m.

A detailed R script is proposed (S1 Script) for r ≤1.

### 2.2 Scaling outbreak size

Until now, outbreak curves have mostly been produced by using a certain explicit outbreak propagation model. This model building is thus considered as a prerequisite, even if the final goal of the simulation is not to understand the mechanisms and factors shaping the curve, nor to implement an explicative model of the outbreak. We adopt a pragmatic, sparing approach by skipping the usual steps of modeling that are required to produce an outbreak curve (S2 Fig). The aim of scaling is to give the outbreak size an intended value while preserving curve shape. We propose that this goal be achieved by testing six Monte Carlo resampling methods.

#### 2.2.1 Inverse transform sampling method (ITSM) for discrete random variables

This method requires that only the form of the density of interest (PMF) and its CDF be known. The random process of sampling needs to create a uniformly-distributed variate *U* in [0,1] for each simulated case. The case dates are found by reading on the CDF the random value (x-axis) corresponding to the variate U on the cumulative probability axis (y-axis). The process is repeated until the expected size of the outbreak is reached.

#### 2.2.2 Binomial method

This method (described in Kachitvichyanukul [33]) makes it possible to simulate independent and identically-distributed (i.i.d.) variables according to an outbreak target distribution (PMF). It considers an outbreak day as a binomial event of the epidemic with a success probability equal to *π*_*i*_ (where *i* is the day of the outbreak). A case is assigned to a day according to the binomial distribution law. This process is repeated until the case sum is equal to the expected size of the outbreak.

#### 2.2.3 Random walk Metropolis-Hastings

A Metropolis-Hastings algorithm [34, 35] is a Markov chain Monte Carlo sampling method that allows the progressive construction of a sample by taking into account a simulated value (*x*_*t-1*_) in order to generate the next one (*x*_*t*_). With Metropolis-Hastings, the Markov chain is built using the original PMF as a stationary density that limits the distribution of the Markov chain [36]. Metropolis-Hastings needs to build a conditional density q(*x*_*t*_|*x*_*t-1*_) (or transition kernel) associated with the original target density (the outbreak curve PMF).

Random Walk Metropolis-Hastings (RWMH) [36] is a practical approach to this method, which carries out a local exploration of the neighborhood of the current value of the Markov chain. The conditional density depends on the previously-simulated value and a random perturbation (*ε*_*t*_):

$${\text{x}}_{\text{t}}={\text{x}}_{\text{t}-1}+\;\varepsilon_{\text{t}}.$$

This random perturbation is the random walk and follows a symmetric distribution (g) centered on zero. The transition kernel between simulated cases is defined by choosing a conditional density:

$$\text{q}\left({\text{x}}_{\text{t}}|{\text{x}}_{\text{t}-1}\right)\;=\text{ g}\left({\text{x}}_{\text{t}}-{\text{ x}}_{\text{t}-1}\right).$$

In our study, *g* followed a uniform distribution and, as proposed by Robert [37], the scale parameter of *ε*_*t*_ was defined as the integer part of the distribution variance.

The simulation process consists in choosing an initial outbreak date and another epidemic day by using the transitional kernel *q(x*_*t*_*|x*_*t-1*_*)*. Adding a case at this new date will be accepted or rejected according to the Hastings ratio.

To set the starting point of the simulation, we used a burn-in period of 1,000 iterations. The same period was used for the other MCMC methods.

#### 2.2.4 Independent Metropolis-Hastings Algorithm (IMHA)

The Independent Metropolis-Hastings Algorithm (IMHA) [35] generates a candidate conditional density that depends only on the present state of the chain.

This density can thus be written: *q(x*_*t*_*|x*_*t-1*_*) = q(x*_*t*_*) = g(x*_*t*_*)*. This method ensures the correlation of the simulated cases.

The process is the same as for RWMH, but the choice of the new date depends on an independent transitional kernel. In our study, the conditional density is a discrete uniform distribution defined over the same range of days as the original PMF.

#### 2.2.5 Gibbs sampling algorithm

This algorithm [36] is used to simulate Markov chain states from a joint distribution *q(x*_*t*_;*y*_*t*_*)*. This requires knowledge of the two conditional distributions *q(x*_*t*_*|y*_*t*_*)* and *q(y*_*t*_*|x*_*t*_*)* that must be built.

We chose slice sampling within the Gibbs sampler approach [38] since it is formally applied with no restriction to any density on its shape or dimension. We consider disease outbreak days (x) and simulated cases (y) as the variables and use a uniform distribution (x, y), which preserves the curve shape despite y changes:

$$p\left(\left(X,Y\right)=\left(x,y\right)\right)=\{\begin{array}{c} \frac{1}{N}\;\;\text{if}\;\;0<y<\pi \left(x\right)\\ 0\;\text{else} \end{array}.$$

According to this joint distribution, we construct the conditional distribution (*q(x*_*t*_*|y*_*t*_*)* and *q(y*_*t*_*|x*_*t*_*)*) and use a Gibbs sampling process.

This sampling consists in selecting an initial date for the simulation and choosing the next case from it by using the conditional density *q(y*_*t*_*|x*_*t*_*)*. We then simulate the next date from this case by using the conditional density *q(x*_*t*_*|y*_*t*_*)*.

A shrinkage procedure is used in this context because we cannot directly simulate a case using *q(x*_*t*_*|y*_*t*_*)*. It consists in randomly choosing a simulated date (x) and testing by means of an interval whether this date can be generated according to the conditional density *q(x*_*t*_*|y*_*t*_*)*.

#### 2.2.6 Hybrid Gibbs sampler (Metropolis-within-Gibbs)

Sometimes the density function obtained from the disease outbreak curve may be unconventional, i.e. discontinuous or resulting from a mixing of different distribution laws (as in the Sverdlosk event or in a measles outbreak). It is difficult for Markov chains to move over discontinuous curves [25]. In theory, the Gibbs sampler should encounter certain difficulties in simulating the shape of this type of curve since some densities cannot be directly simulated. A hybrid Gibbs sampler, in the Metropolis-within-Gibbs strategy sense, may be a solution [39]. The idea is to use a preliminary step consisting of a random walk simulation of Markov chain states instead of a direct simulation within Gibbs sampling. Hybrid strategy associates the conditional density *q(x*_*t*_*|y*_*t*_*)* with the Hastings ratio, allowing the exploration of all the outbreak curves.

### 2.3 Quality assessment

When the duration or the number of cases during an outbreak simulation is changed, the shape of the outbreak (representing a latent process) must be maintained within a range of plausible epidemiological curves, which could be defined as the range of real triplets (size, duration, disease) observed during real outbreaks (obtained from publications). This necessity is also true for mechanistic or statistical distribution-based methods that may also face the risk of simulating an unrealistic outbreak.

The same problem was highlighted by the High-Fidelity Injection Detectability Experiments (HiFIDE) project in 2005. It tried to extend the semi-synthetic method by conserving the relationship between "the magnitude of the real outbreak and the strength of the signal in the surveillance data collected during the real outbreak" [9]. Without forgetting this simulation constraint during a real evaluation, in a first theoretical approach (proof of concept) with the aim of testing the capacity and the limits of MC methods, we chose to generate each triplet resulting from the combination of 12 sizes (10, 20, 30, 50, 100, 300, 500, 1000, 2000, 3000, 4000 and 5000 cases), six durations (5, 7, 14, 28, 42, 56 days) and four real outbreak shapes (Norovirus, Measles, Anthrax, Dengue) [24, 40–42]. We replicated each simulation 20 times and used a median for evaluating stochastic algorithm performance, as proposed by Birattari [43].

Since all the methods proposed in this study are able to precisely simulate duration and size, quality assessment will be based only on the degree of conformity to the real outbreak shape.

#### 2.3.1 Goodness-of-fit (GOF)

Curve shape preservation was evaluated using the distance between the original real curve and the simulated one. We chose a GOF approach to evaluate this distance considered to be an indicator of simulation quality.

A Goodness-of-Fit (GOF) test between two distributions is usually indicated to determine whether an observed distribution corresponds to a particular probability distribution (such as the historical outbreak distribution). Due to the constraints of the test approach (e.g. the necessity to define a significance level α that varies with the quantity of simulated data), we chose to use GOF metrics (distance, divergence, similarity) as proposed by several authors [44, 45] to compare simulation qualities.

There is currently no consensus in the literature on the choice of a metric. Moreover, since there is no certainty regarding the convergence of quality results according to different metrics, we selected a limited number of metrics corresponding to different GOF properties. Due to the discrete nature of data, we selected the L2-distance built for comparing distance between two densities. Pearson divergence and Kolmogorov distance were chosen since they are derived from the statistics used to evaluate GOF between two probability density functions. And to evaluate similarity, we chose the Overlap (delta) Coefficient and Matusita’s (Rho) Measure [44, 45]. All the GOF metrics are detailed in the S1a and S1b Table.

To avoid scale effects, all metrics were normalized. A two-sample T-test was used to compare the metrics between two groups.

#### 2.3.2 Overall scale factor

Simulation relies on a scaling process. We assumed this scaling process could affect the quality of results, and in particular lead to a possible loss of information resulting from scattering a small number of cases over the duration of the outbreak.

To estimate the influence of the scaling process on simulations, we first calculated the case density for each distribution as the number of cases divided by the number of days. We then calculated the scale factor as the case density ratio of the simulation divided by the case density of the original distribution. During our work, we studied the impact of this scale factor on the quality of the simulations evaluated by different metrics.

## 3 Results and discussion

We first identified the input parameters that contribute most to the accuracy of results and investigated the way a change of input parameters influences results (Table 1).

**Table 1:** Evaluation of six resampling methods according to outbreak type, number of cases and number of days simulated.

Outbreak	Measles	Anthrax	Dengue	Norovirus		
Binomial	0.025* *(0*.*00)*^†^	0.033 *(0*.*00)*	0.042 *(0*.*00)*	0.036 *(0*.*00)*		
ITSM	0.027 *(0*.*64)*	0.033 *(0*.*70)*	0.042 *(0*.*73)*	0.041 *(0*.*99)*		
Metrop. RW	0.082 *(0*.*53)*	0.071(0.57)	0.086 *(0*.*58)*	0.062 *(0*.*81)*		
Metrop. Ind	0.085 *(0*.*54)*	0.089 *(0*.*59)*	0.080 *(0*.*64)*	0.097(0.80)		
Gibbs	0.037 *(0*.*77)*	0.039 *(0*.*70)*	0.045 *(0*.*66)*	0.038 *(0*.*95)*		
Hybrid	0.083 *(0*.*81)*	0.088 *(0*.*90)*	0.075 *(1*.*04)*	0.096 *(1*.*00)*		
Cases	10	50	100	500	1000	5000
Binomial	0.185 (0.00)	0.038 (0.00)	0.020 (0.00)	0.004 (0.00)	0.002 (0.00)	0.000 (0.00)
ITSM	0.190 (0.00)	0.043 (0.03)	0.020 (0.06)	0.004 (0.28)	0.002 (0.57)	0.000 (2.87)
Metrop. RW	0.327 (0.21)	0.099 (0.23)	0.060 (0.24)	0.015 (0.36)	0.008 (0.51)	0.002 (1.77)
Metrop. Ind	0.411 (0.22)	0.107 (0.23)	0.056 (0.25)	0.011 (0.38)	0.006 (0.53)	0.001 (1.82)
Gibbs	0.201 (0.26)	0.047 (0.28)	0.024 (0.29)	0.005 (0.44)	0.003 (0.62)	0.000 (2.23)
Hybrid	0.401 (0.34)	0.104 (0.35)	0.057(0.38)	0.011 (0.55)	0.005 (0.77)	0.001 (2.65)
Days	5	14	28	42	56	
Binomial	0.005 (0.00)	0.018 (0.00)	0.036 (0.00)	0.057 (0.00)	0.081 (0.00)	
ITSM	0.006 (0.64)	0.021(0.71)	0.039 (0.77)	0.057 (0.87)	0.085 (0.93)	
Metrop. RW	0.016 (0.60)	0.046 (0.62)	0.088 (0.60)	0.124 (0.63)	0.155 (0.65)	
Metrop. Ind	0.011 (0.65)	0.046 (0.65)	0.096 (0.63)	0.156 (0.64)	0.199 (0.64)	
Gibbs	0.008 (0.72)	0.024 (0.78)	0.048 (0.81)	0.058 (0.83)	0.087 (0.74)	
Hybrid	0.011 (0.78)	0.046 (0.86)	0.092 (0.96)	0.155 (1.12)	0.190 (1.13)	

*Normalized Pearson distance averaged for all other parameters

^†^ (Average time of simulation in seconds)

Binomial = Binomial method / ITSM = Inverse transform sampling method / Metrop. RW = Metropolis-Hasting Random Walk /Metrop. Ind = Metropolis-Hasting independent sampler / Hybrid = Hybrid Gibbs sampler / Gibbs = Gibbs sampling algorithm

### 3.1 The influence of outbreak duration and size

**Fig 2:**
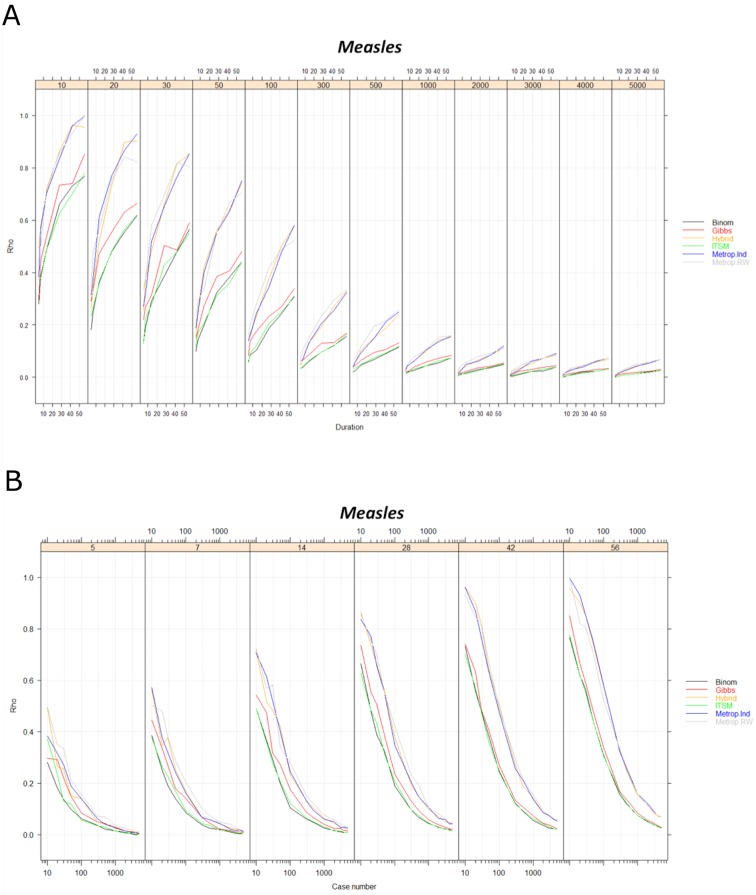
Measles outbreak simulation assessment. (a) according to the outbreak duration (in days). (b) according to the number of simulated cases. Rho: Matusita’s Measure used for evaluation / Binom = Binomial method / ITSM = Inverse transform sampling method / Metrop. RW = Metropolis-Hasting Random Walk / Metrop. Ind = Metropolis-Hasting independent sampler / Hybrid = Hybrid Gibbs sampler / Gibbs = Gibbs sampling algorithm.

Quality decreases with the duration of an outbreak and increases with its size for all combinations of curve, algorithm and metric chosen for the evaluation. An example for a measles outbreak is presented in Fig 2. Two groups of algorithms (the Binomial, ITSM and Gibbs group versus the Metropolis Algorithms and Hybrid group) can be distinguished based on their sensitivity to the outbreak duration. The first group seems less influenced by a change in duration (Fig 2a). The GOF difference (normalized Pearson metric) is significant between these two different groups (p<0,001).

Rho distance for the difference of inadequacy between binomial and independent Metropolis algorithms averages 11% [min = 0.5%—max = 31%] (Fig 2b). The maximum difference between the two groups was observed for outbreaks of 20 cases, with an average difference of 24%. Difference increases continuously and linearly with outbreak duration.

Simulation quality increases exponentially (R^2^ > 0.97) with the number of cases (Fig 2b) and with decreasing outbreak duration.

**Fig 3:**
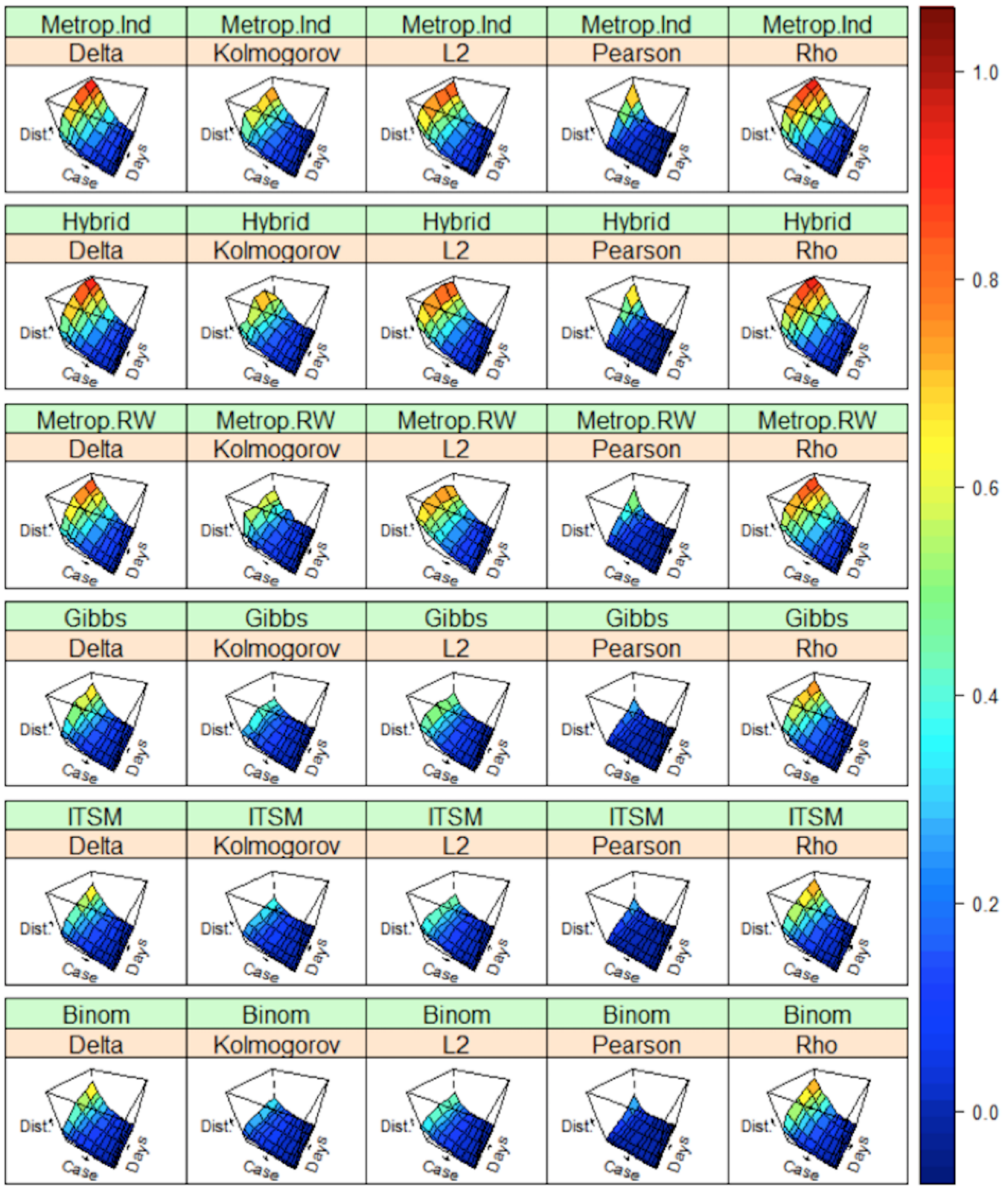
Average quality (between the generated outbreak curves and the corresponding historical epidemic curve) for all diseases considered. **Quality was evaluated by different metrics, according to the number of cases and duration**. Dist. = normalized distance used for evaluation detailed in pink line; Delta = Overlap Coefficient; Kolmogorov = Kolmogorov distance;; L2 = L2-distance; Pearson = Pearson distance, Rho = Matusita’s Measure; Technique used for simulation in green line: Binom = Binomial Method / ITSM = Inverse Transform Sampling Method / Metrop. RW = Metropolis-Hasting Random Walk /Metrop.Ind = Metropolis-Hasting Independent Sampler / Hybrid = Hybrid Gibbs Sampler / Gibbs = Gibbs Sampling Algorithm.

With regard to the interaction between size and duration, the Metropolis-Hasting independent sampler and Hybrid Gibbs sampler are nearly equivalent in terms of accuracy. Random Walk Metropolis-Hasting performs slightly better in extreme simulation values. The real gap appears with Gibbs sampling, followed by ITSM and binomial methods for all simulations (Fig 3) and curve shapes.

### 3.2 Comparison of algorithms

This work examines how to simulate realistic infectious disease outbreak curves with a minimum number of parameters. As for any temporal simulation, we needed to provide two parameters: duration and size, and as is the case for a simple mathematical distribution approach, we had to choose a distribution shape among a set of real outbreaks. The resampling methods we tested, especially the binomial method and ITSM, are easy to implement even for a non-specialist. Although all these methods are able to generate a simulated curve from real outbreaks, their accuracy is algorithm-dependent.

Simulation quality for all diseases considered was measured (using different Goodness-of-fit measures) by calculating the difference between the generated outbreak curve and its corresponding historical outbreak curve while taking into account the influence of the number of cases and duration of the outbreak simulated. In Fig 3, for a given GOF, the lower the surface (greens and blues) in the cube, the better the quality of the simulation. We can observe that algorithms based on MCMC are less precise, regardless of the GOF chosen, than those based on independent sampling (Table 1, Fig 3).

The binomial algorithm had the best performance and was fast, since we implemented it using Rbinom, an optimized function based on C language directly implemented in the core package of R.

While generally accurate and fast, the ITSM algorithm was slower (Table 1) than most other algorithms with increasing size or duration of the simulated curve. In theory, the "burn-in" process penalizes all MCMC algorithms, but when the number of cases and days increases, the study shows that ITSM takes twice as much time as RWMH. In fact, ITSM is slower than other methods [46], especially for a large sample, due to the number of comparisons it requires. However, this method can be used for any type of distribution function, including a mixture of discrete distributions.

Since an outbreak curve may have several local minima/maxima and regions of low probability, a Markov chain may fail to explore all of this complex distribution, except for Gibbs-based methods. For dependent variables, the convergence of Markov chains with any particular distribution is not guaranteed [47].

Gibbs sampling with a shrinkage procedure is designed to manage complex distributions [36]. This two-stage Gibbs sampler ensures the convergence of the Markov chain [36]. The hybrid algorithm and Metropolis group has an acceptance rate of less than 1, thus convergence cannot be ensured or is reached more slowly than for a Gibbs algorithm. This probably explains the difference between Gibbs and the other MCMC algorithms. The accuracy of the Gibbs algorithm was close to the independent (i.i.d.) algorithms, but Gibbs has a slower convergence. In theory, Gibbs produces samples of disease outbreak distributions that are more realistic, reproducing the temporal correlation of data.

The proposal distribution has a strong influence on IMHA performance [36], which is problematic with the complexity of outbreak curves. For example, this algorithm performed best with dengue outbreaks, which have a curve that resembles a uniform distribution.

Random Walk Metropolis-Hastings (RWMH) often appears to be a generic algorithm. But a frequent issue of RWMH is whether or not the random walk should explore all the regions of the distribution to be sampled. Robert [36] explains that RWMH "usually is not necessarily the most efficient choice because it requires many iterations to overcome difficulties (such as low-probability regions of curve) and because of its symmetric features, it spends roughly half the simulation time revisiting regions it has already explored". Our study confirms that RWMH is more efficient for simulating a symmetric jumping distribution, such as that of the norovirus outbreak, than other asymmetric distributions.

The fundamental advantage offered by a Metropolis-within-Gibbs (hybrid) structure is that it breaks down a complex curve into a number of smaller and simpler targets, where local MH algorithms can be tailored at little cost. While these algorithms are very successful for many target distributions, they cannot work efficiently when many wasteful jumps or small moves are generated. The phenomenon is generally encountered in high-dimensional cases or some low-dimensional extreme cases [48]. The hybrid algorithm performed best for dengue and measles outbreaks (outbreaks with non-connected zones), probably because of the ability of this method to explore these types of complex curves.

### 3.3 Influence of the overall scale factor

**Fig 4:**
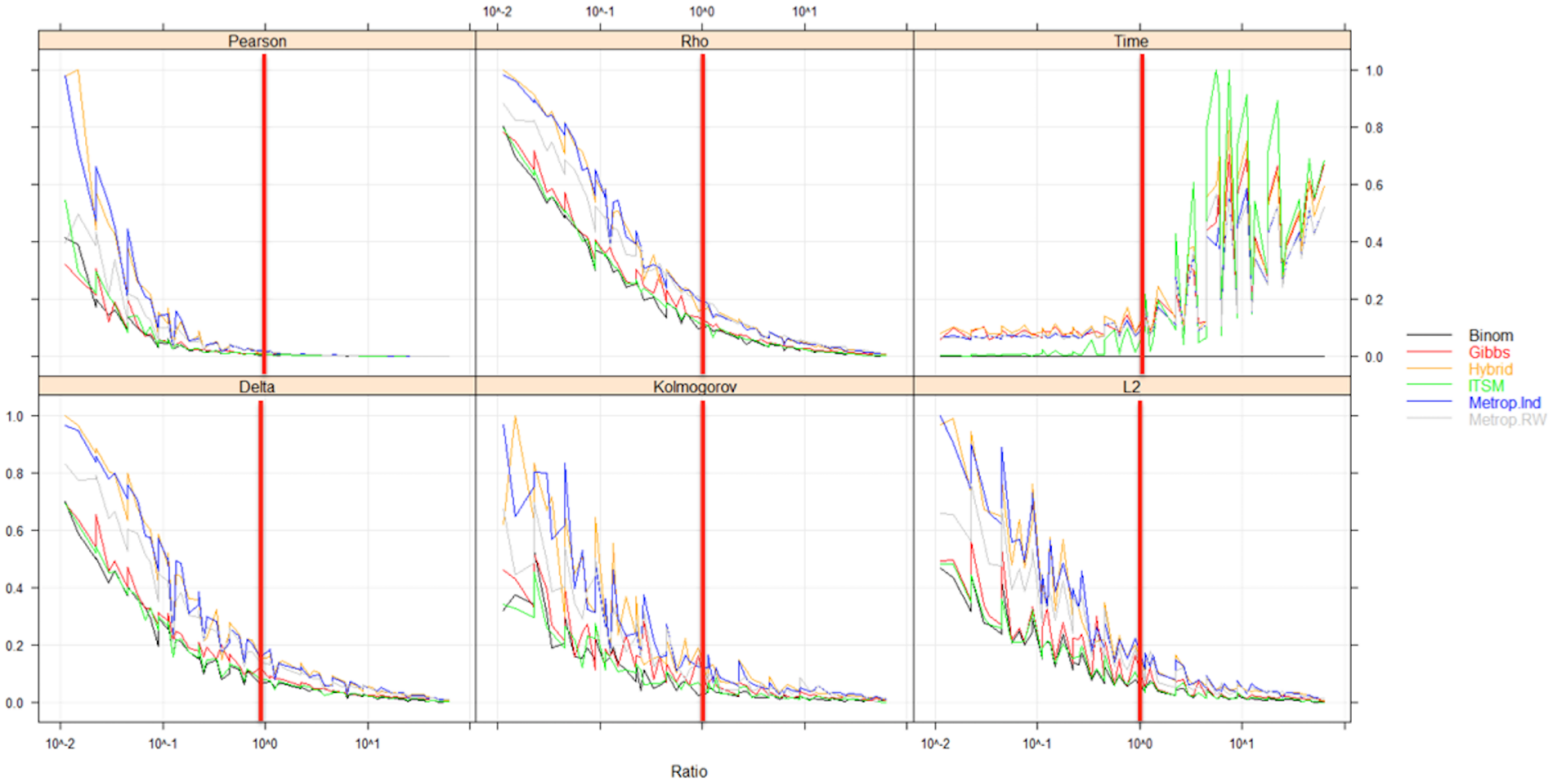
Algorithm simulation accuracy measured by goodness-of-fit measures* and time according to the ratio of simulation. *Delta = Overlap Coefficient, Kolmogorov = Kolmogorov distance; L2 = L2-distance, Pearson = Pearson distance, Rho = Matusita’s Measure.

The overall scale factor (OSF) describes how outbreak size and duration interact by comparing the original and resulting curves. The results of the OSF study are presented in Fig 4.

When the OSF is below 1 (i.e. when the number of cases per day in the simulation is less than that of the original curve), the accuracy and realism of the simulation, evaluated by GOF metrics, decrease rapidly (significant difference, p<0,001). For example, the median loss of accuracy below this threshold is equal to 14.6% for the binomial method (15.7% for ITSM, 16.1% for Gibbs) and 43.8% for Metropolis Independent (42.3% for hybrid, 31.2% for RWMH). Over 1 and for the same algorithms, the loss of accuracy is respectively 0.22% (0.23% for ITSM, 0.29% for Gibbs) and 0.63% (0.64% for hybrid, 1.0% for RWMH). The accuracy is 2 to 3 times better for the first group of algorithms. Moreover, an OSF below 1 is associated with an increase in the variance and duration of the simulation.

In fact, an overall scale factor (OSF) below 1 is associated with a significant loss of information during the simulation process. Regardless of the outbreak simulated, OSF = 1 can be considered as a kind of threshold below which the loss of information increasingly impairs simulation quality in terms of goodness-of-fit and variance. As an overall measure of simulation GOF, we used and recommend the χ^2^-divergence, which is also a measure of the loss of information during the simulation process [49, 50].

### 3.4 Final considerations

The purpose of our study is not to evaluate existing algorithms but to propose a simple way to generate realistic and controlled (with regard to the most important characteristics influencing algorithm evaluation) outbreak signals. This preliminary step is required in order to carry out reproducible evaluations expected by system designers/practitioners and to evaluate the influence of the shape and the pathogen on the ODA performance. Our approach follows a pragmatic principle of parsimony by skipping the usual steps of modeling required to produce an outbreak curve (S2 Fig). Indeed, resampling methods are often used as a robust alternative to those based on parametric assumptions when they are dubious, or when parametric inference is impossible or requires very complex formulas for computation. Another advantage of resampling methods resides in the fact that our goal can be achieved without generating and validating a plausible model of transmission and surveillance.

The strength of Monte Carlo resampling methods is their ability to produce realistic and customized curves by rebuilding the original underlying distribution directly from a single observed curve (the epicurve probability distribution), even if the outbreak curve is unusual, provided that the outbreak PMF (probability mass function) can be extracted from real data. Creating a database of outbreak curves (Disease or shape curve/Number of cases/Number of days) supplemented with contextual information (such as population under surveillance/time/location) can provide the material for various simulations and may help to define what constitutes a realistic and plausible epidemiological curve faced by the surveillance system. In this study, we present only four shapes for a proof of concept, but our database is currently made up of 893 real outbreaks resulting from 58 diseases under surveillance, allowing the generation of many other curves. However, the quality (accuracy) of the choice of curve to support the resampling process during the simulation is clearly the major limitation of the method.

As for all simulation approaches, an evaluation of the epidemiological situation (population, agent, system, location, period, etc.) of the original observed curve/model is required when choosing the one that will support the simulation. Until our proposal, simulation of the complex shape of the measles, dengue or anthrax outbreaks (presented in Fig 1) for evaluating a disease surveillance system and ODA was based on the curve/shape set presented in S1 Fig, which is considered the reference [6, 7, 16–19] for simulating outbreaks with controlled size and duration. The strength of resampling methods is their ability to preserve as much as possible the general shape of real outbreak curves (see an example provided in S3 Fig), which is known to influence the results of ODA evaluations. However, it is clear that one limitation of our study is the difficulty of proving that each produced curve is realistic. Ideally, each curve should be compared to curves observed during real surveillance activity, capturing all specific patterns of the outbreak observed, and not only to the published curve (considered as the reference curve) as in our case.

Our approach can be considered as an easier method than implementing a propagation model or using existing code from compartmental epidemic models, which require numerous real parameters. For example, in the case of the binomial method (the simplest method used in our study), a simulation of an outbreak of 30 cases over 10 days assigns each new case to one of the 10 days according to the binomial distribution law (the PMF extracted from the real curve providing the probability of success on each trial). The corresponding r code is rbinom (number of days, number of cases, probability mass function vector extracted from a real curve) or in our example rbinom(10, 30, PMF). This code is the same, irrespective of the disease outbreak.

## 4 Conclusion

Given the constraint of keeping the simulation within a range of plausible epidemiological curves faced by the surveillance system, our study confirms that MC resampling methods applied to historical disease outbreaks can be used for outbreak curve production. It also confirms the influence of the algorithm and outbreak duration, size and curve shape on simulation quality. It suggests that Gibbs sampling with a shrinkage procedure is a convenient approach to simulating outbreak curves, since it can be viewed as a fair trade-off between accuracy, simulation speed and data dependency. If dependency is of little importance, binomial and ITSM methods are accurate. This study also shows that χ^2^-divergence is a useful tool, not only for evaluating simulation quality but also for quantifying the loss of information observed throughout the simulation process.

Finally, we confirm that the signal generation method proposed in this work must be able to simulate a set of signals for evaluation simply, including very unlikely ones (for testing special or extreme situations). Even if our approach can generate a large spectrum of possible signals, we do not find it necessary to test algorithm performance on all these unlikely signals, but only on the authentic signals faced by the surveillance system.

## Supporting information

S1 Script Function Dayscale (in R language) for homogeneous dilation of number of time steps in the case of a scaling factor r≤1 (number of time steps simulated superior to the number of time steps in the initial curve). (DOCX)

S1 Fig Canonical shapes of simulated outbreaks generally used for ODA evaluation. (TIF)

S2 Fig Comparison between different approaches used to generate outbreak curves. (TIF)

S3 Fig Example of simulation and evaluation process in the case of a measles outbreak shape [40], for a 14-day duration and 30-case outbreak simulation. (TIF)

S1 Table Goodness of fit metrics. (a) Distance and divergence measures used for evaluation. (b) Measure of similarity used for evaluation. with P_i_ and P_i0_: Probability Mass Function to compare.
(DOCX)
