# Supplementary material for: Building test data from real outbreaks for evaluating detection algorithms
Source: PLoS One. 2017 Sep 1;12(9):e0183992. doi: 10.1371/journal.pone.0183992 (PMC5593515; doi:10.1371/journal.pone.0183992)
Supplement: S1 Script — (DOCX) [file pone.0183992.s001.docx]

**Script S1:** Function Dayscale (in R language) for homogeneous dilation of number of time steps in the case of a scaling factor r≤1 (number of time steps simulated superior to the number of time steps in the initial curve)

*# Example of PMF (Probability mass function) used for data input: Norovirus outbreak (Alaska, 1999) # during 12 time steps (Centers for Disease Control and Prevention 2000)*

*# >data*

*# epidName epidTime caseProb*

*# Norovirus 1 0.005235602*

*# Norovirus 2 0.010471204*

*# Norovirus 3 0.026178011*

*# Norovirus 4 0.015706806*

*# Norovirus 5 0.178010471*

*# Norovirus 6 0.445026178*

*# Norovirus 7 0.193717278*

*# Norovirus 8 0.062827225*

*# Norovirus 9 0.015706806*

*# Norovirus 10 0.036649215*

*# Norovirus 11 0.005235602*

*# Norovirus 12 0.005235602*

*# Function Dayscale (building a band matrix allowing homogeneous dilation of number of time steps)*

Dayscale <- function(data,m) { *# m = number of time steps to simulate*

n <- dim(data)[1] *# n = number of time steps i in the initial outbreak (ex : 12)*

r <- n/m *# r = scale factor, default value for fij*

i <- 1 *# column index (initial vector)*

j <- 1 *# line index (final vector)*

SP <- 0 *# f_ij_ sum initialisation*

P <- c(rep(0,m)) *# f_j_ vector: fraction of π_i_ at time graduations i participating to Pj*

while ((i <= n) && (j <= m)) {

if (P[j]==0) {

if ((SP+r) <= 1) {

P[j] <- r*data$caseProb[i] *# Pj = Final probability at step j*

SP <- SP + r *# fij summation*

j <- j+1 *# next final distribution step*

}

else {

a <- (1-SP) *#* *a = proportion of time graduations i not used to create step j*

P[j] <- a*data$caseProb[i] *#* *attribution of quota a of initial probability*

SP <- 0 *# sum reinitialisation*

i <- i+1 *# next initial distribution step*

}

}

else { *# there is a remain to attribute*

a <- r-a *# remain to attribute*

SP <- a *# sum reinitialisation*

P[j] <- P[j]+a*data$caseProb[i] *#* *attribution of quota a of initial probability*

j <- j+1 *# next final distribution step*

}

}

*# Daynew = final table after simulation with new PMF and CDF*

epidNameN<-rep(data$epidName[1],m)

Daynew<- as.data.frame (cbind(as.factor (epidNameN), as.integer (c(1:m)), P, cumsum(P)))

names(Daynew)<-c("epidName","epidTime","caseProb")

Daynew$epidName<-as.factor(epidNameN)

print (Daynew)

return (Daynew)

}
