# Supplementary figures and images for: Building test data from real outbreaks for evaluating detection algorithms
Source: PLoS One. 2017 Sep 1;12(9):e0183992. doi: 10.1371/journal.pone.0183992 (PMC5593515; doi:10.1371/journal.pone.0183992)

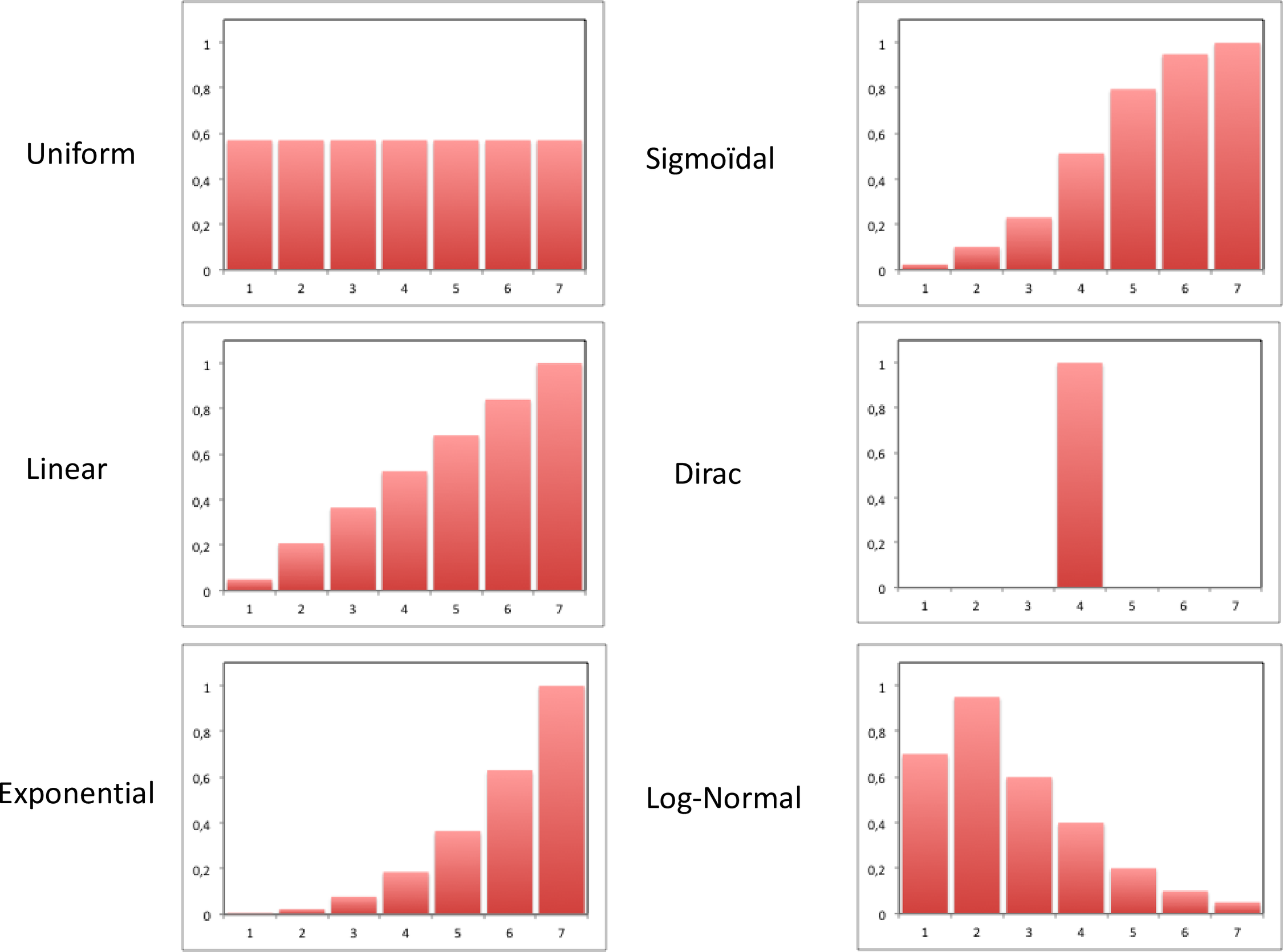

Supplement: S1 Fig — (TIF) [file pone.0183992.s002.tif]

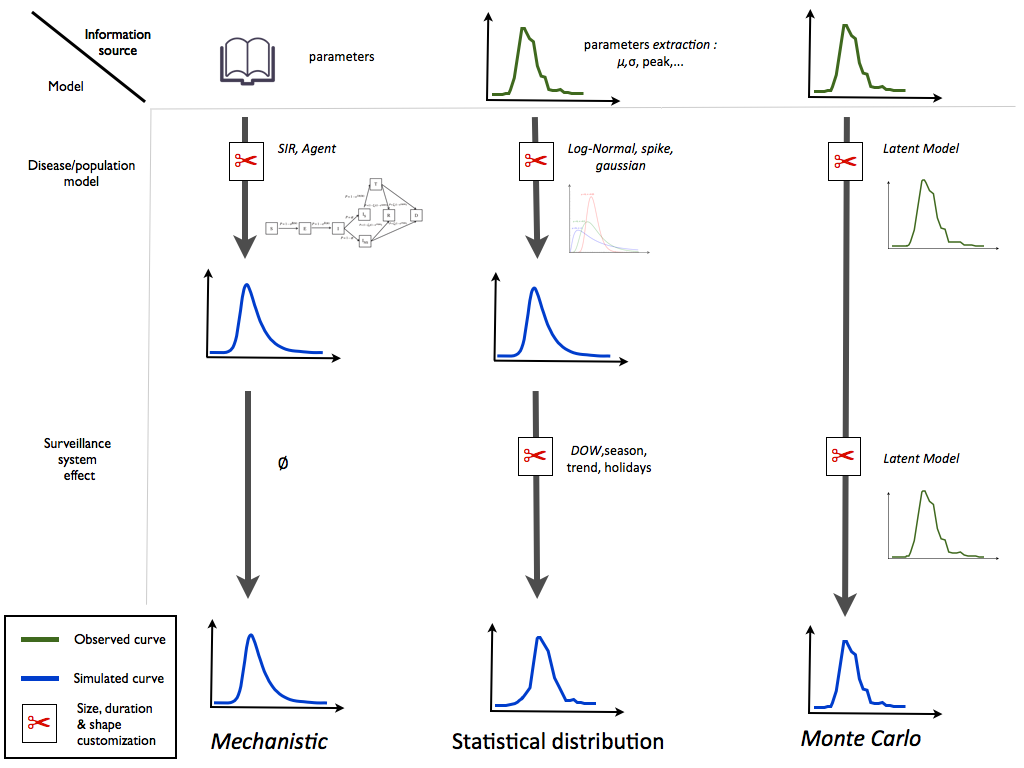

Supplement: S2 Fig — (TIF) [file pone.0183992.s003.tif]

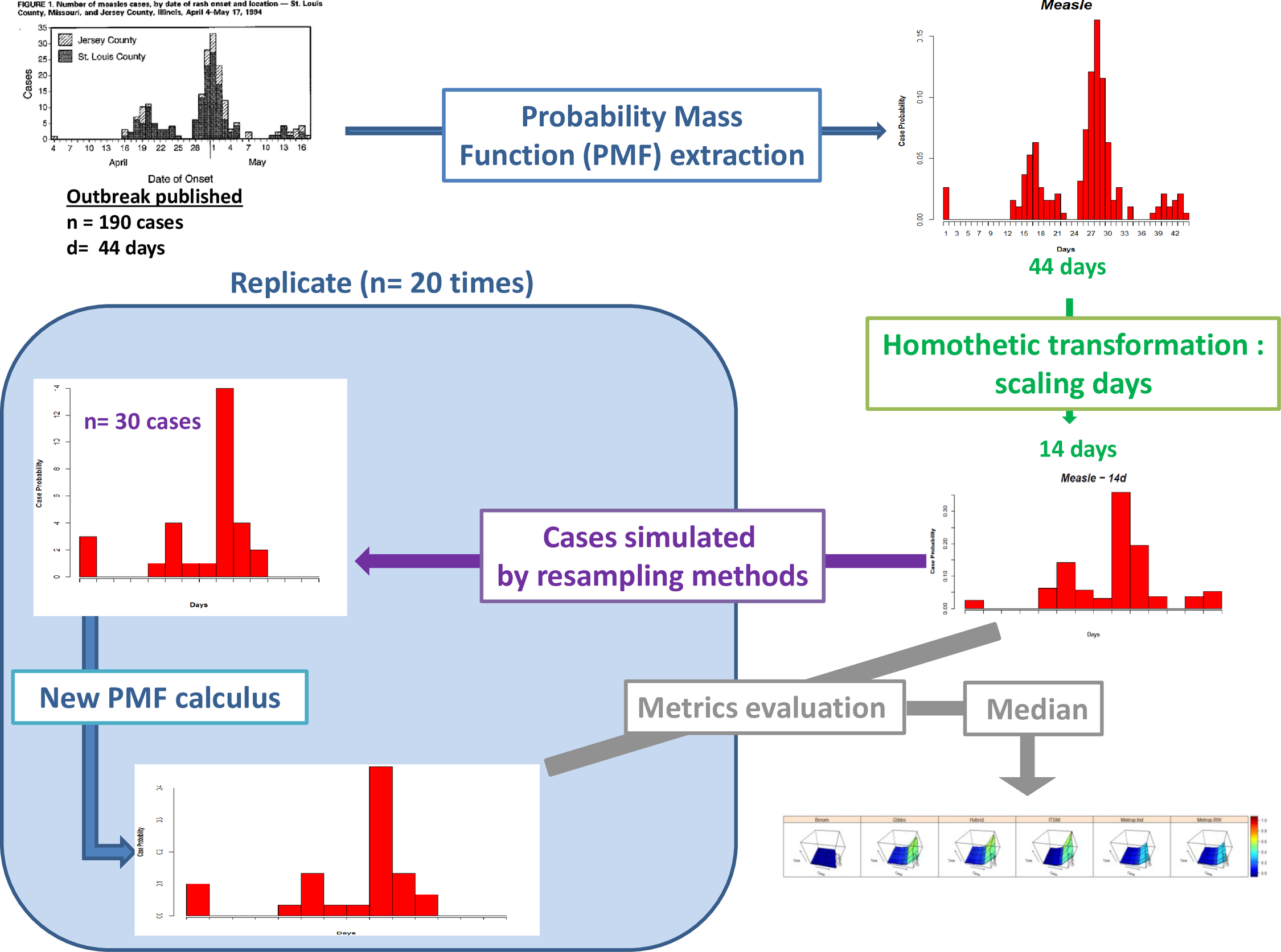

Supplement: S3 Fig — (TIF) [file pone.0183992.s004.tif]
