# Supplementary material for: Building test data from real outbreaks for evaluating detection algorithms
Source: PLoS One. 2017 Sep 1;12(9):e0183992. doi: 10.1371/journal.pone.0183992 (PMC5593515; doi:10.1371/journal.pone.0183992)
Supplement: S1 Table — (a) Distance and divergence measures used for evaluation. (b) Measure of similarity used for evaluation. with Pi and Pi0: Probability Mass Function to compare. (DOCX) [file pone.0183992.s005.docx]

**Table S1. Goodness of fit metrics** (with P_i_ and P_i0_ : Probability Mass Function to compare)

Table S1a: Distance and divergence measures used for evaluation

| Metrics | Formula |
| --- | --- |
|  |  |
| L2-distance |  |
| Kolmogorov |  |
| Pearson |  |
|  |  |

Table S1b : Measure of similarity used for evaluation

| Metrics | Formula |
| --- | --- |
| Overlap (delta) Coefficient |  |
| Matusita’s Measure |  |
|  |  |
